# Invasive adenocarcinoma associated with intraductal tubulopapillary neoplasm in young adult mimicking alcoholic pancreatitis and pseudocysts

**DOI:** 10.1093/jscr/rjag558

**Published:** 2026-07-10

**Authors:** David Nolte, Ifeomachukwu E Nwosu, Hina Arif, Mohammad Khreiss, Achyut K Bhattacharyya, Belinda L Sun

**Affiliations:** Department of Pathology, Banner-University Medical Center, College of Medicine, University of Arizona, 1625 N Campbell Ave, Tucson, AZ 85721, United States; Department of Pathology, Banner-University Medical Center, College of Medicine, University of Arizona, 1625 N Campbell Ave, Tucson, AZ 85721, United States; Department of Radiology and Imaging Sciences, Banner-University Medical Center, College of Medicine, University of Arizona, 1625 N Campbell Ave, Tucson, AZ 85721, United States; Department of Surgery, Banner-University Medical Center, College of Medicine, University of Arizona, 1625 N Campbell Ave, Tucson, AZ 85721, United States; Department of Pathology, Banner-University Medical Center, College of Medicine, University of Arizona, 1625 N Campbell Ave, Tucson, AZ 85721, United States; Department of Pathology, Banner-University Medical Center, College of Medicine, University of Arizona, 1625 N Campbell Ave, Tucson, AZ 85721, United States

**Keywords:** intraductal tubulopapillary neoplasm, intraductal tubulopapillary neoplasm with associated carcinoma, alcoholic pancreatitis, pancreatic pseudocyst

## Abstract

Intraductal tubulopapillary neoplasm (ITPN) of the pancreas is a rare tumor that is often difficult to diagnose preoperatively because of nonspecific symptoms and atypical imaging findings. Accurate identification is important, as ITPN carries a significant risk of associated invasive carcinoma. We report a case of ITPN with carcinoma in a 30-year-old man who presented with recurrent alcoholic pancreatitis, pancreatic pseudocysts, and a pancreatic duct stricture. Distal pancreatectomy revealed ITPN with invasive carcinoma. He received adjuvant FOLFIRINOX, but developed locally recurrent adenocarcinoma 17 months later, requiring completion total pancreatectomy. Postoperative therapy included chemoradiation with gemcitabine, nab-paclitaxel, and capecitabine, and on maintenance gemcitabine two years after the second surgery. This case illustrates the diagnostic and therapeutic challenges of ITPN in young adults and underscores the need to distinguish it from more common pancreatic diseases. Notably, carcinoma arising from ITPN may have a more favorable prognosis than conventional pancreatic ductal adenocarcinoma.

## Introduction

Intraductal tubulopapillary neoplasm (ITPN) is a rare pancreatic neoplasm, accounting for less than 1% of all exocrine pancreatic tumors and approximately 3% of intraductal neoplasms [1]. ITPN was initially described by Yamaguchi *et al.* [1] in 2009 and subsequently recognized as a distinct entity in the World Health Organization (WHO) Classification of Tumours of the Digestive System, fourth edition (2010) and reaffirmed in the fifth edition (2020) [2, 3]. Pathologically, ITPN is defined as an intraductal, predominantly tubule-forming epithelial neoplasm with high-grade dysplasia and ductal differentiation, without overt mucin production [2, 3]. Importantly, ITPN carries a high risk of malignant transformation, with associated carcinoma reported in up to 71% of cases [4]. Unlike more common pancreatic tumors such as intraductal papillary mucinous neoplasms (IPMN) or conventional ductal adenocarcinoma, which typically occur in older individuals, ITPN can present in younger adults, with the youngest reported case at 25 years of age [4]. Patients with ITPN often present with nonspecific clinical symptoms, and some cases are discovered incidentally [4]. Preoperative diagnosis remains challenging, particularly in younger patients, due to atypical symptoms and variable radiologic manifestations across imaging modalities [5]. Nevertheless, awareness of this rare entity and its atypical clinical features is crucial, as accurate diagnosis is essential for optimal management given the high association with invasive carcinoma. Here, we report a case of ITPN with associated invasive adenocarcinoma in a 30-year-old male, initially misdiagnosed as chronic alcoholic pancreatitis with pseudocyst formation prior to surgical treatment for recurrent pseudocysts and pancreatic duct stricture.

## Case report

### Clinical history and imaging

**Figure 1:**
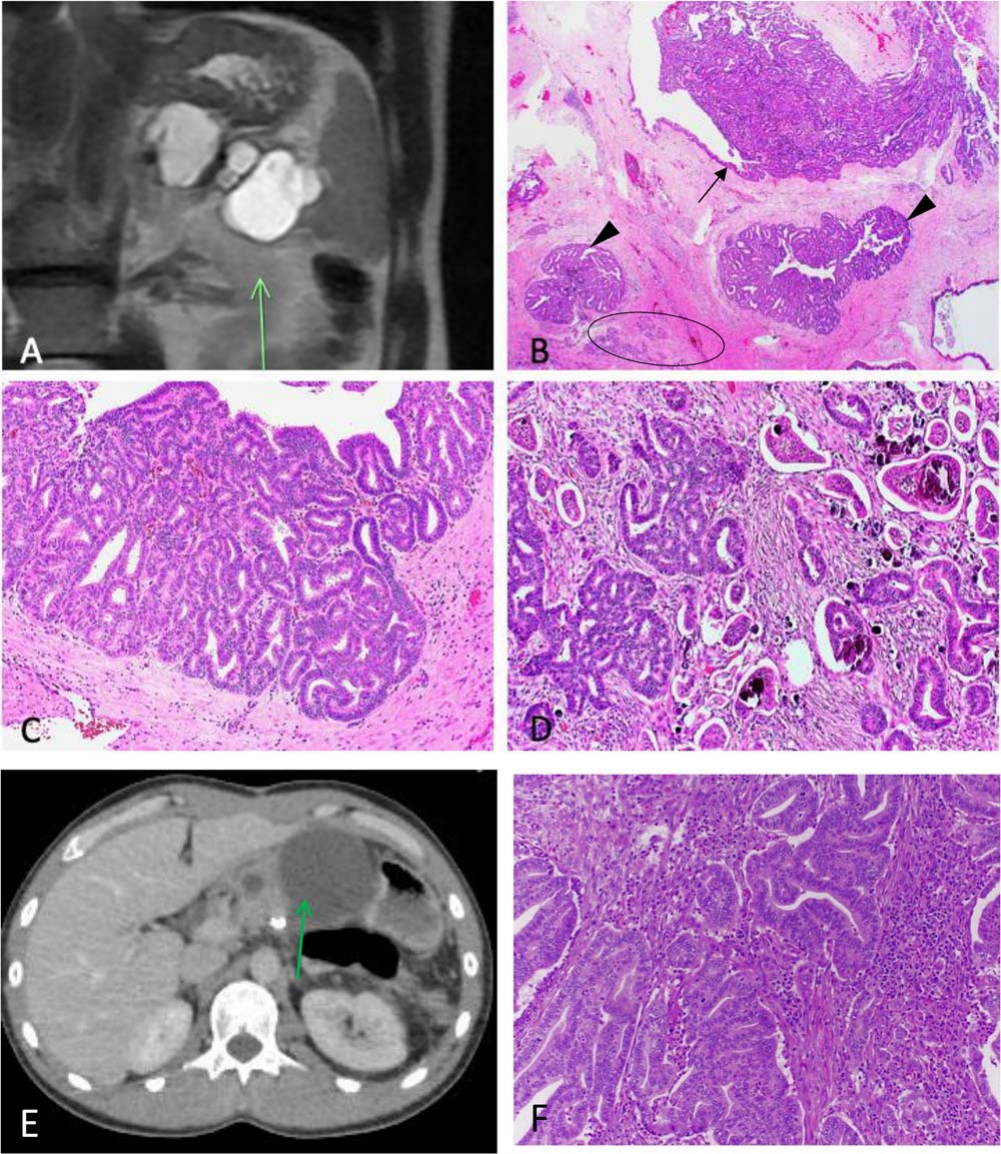
Carcinoma associated with ITPN. (A) T2 SPAIR MRI imaging shows pancreatic multicystic complex lesion (arrow) with heterogenous components in pancreas. (B) Low-power magnification microscopic examination shows an ITPN within the main pancreas duct (arrow) and branch ducts (arrowheads), and an area of invasive carcinoma (circle) in a background of chronic pancreatitis. 50×. (C) The ITPN is composed of back-to-back tubules and papillae without mucin. 200×. (D) The area with invasive adenocarcinoma shows cribriform complex glands, angulated or micropapillary invasive glands with desmoplastic stromal reaction, and tumor calcifications. 200×. (E) Computed tomography imaging shows large cystic lesion (arrow) at the previous resection margin. F: The recurrent tumor shows invasive complex glands with background acute inflammation.

A 30-year-old man with a history of alcohol use and recurrent pancreatitis presented with nausea and abdominal pain. The patient was initially evaluated at an outside institution, where outside hospital imaging revealed multiple pancreatic cystic lesions, the largest measuring 2.4 cm in the body, with distal ductal dilation. The findings were initially interpreted as pseudocysts related to chronic pancreatitis, and the patient underwent pancreatic duct stenting and cystogastrostomy. No tissue or fluid samples were sent for pathology or fluid analysis at outside hospital. The largest cyst decreased to 1.8 cm, and the device was removed. Eight months later, follow-up imaging demonstrated a pancreatic duct stricture with upstream dilation (6 mm) and recurrent cysts up to 3.5 cm (Fig. 1A). Given the recurrent cysts and ductal stricture, a distal pancreatectomy was performed without attempting preoperative biopsy or cyst fluid analysis.

### Gross and histopathologic findings

The distal pancreatectomy specimen revealed a 6.3 cm cystic (60% of lesion) and 3.5 cm solid (40% of lesion) lesion centered along the pancreatic duct and approaching the resection margin. Microscopically, the lesion consisted of a tubulopapillary epithelial neoplasm primarily within the main duct (Fig. 1B), with obliteration of branch ducts and complex tubular gland architecture showing high-grade dysplasia (Fig. 1C). Tumor cells were cuboidal with hyperchromatic nuclei and high nuclear-to-cytoplasmic ratios; mitotic figures were frequent. No prominent mucin production was identified. While MUC1 and MUC6 positivity and MUC2/MUC5AC negativity are commonly reported immunoprofiles in ITPN, the diagnosis of ITPN in the present case was supported despite the absence of these immunohistochemical studies. The diagnosis was based on the characteristic histomorphologic findings, including the predominant tubulopapillary growth pattern, high-grade cytology, intraductal localization, and relative absence of overt mucin production. Furthermore, the morphologic features in this case were not supportive of IPMN or solid-pseudopapillary neoplasm. Associated invasive carcinoma displayed angulated and cribriform glands in desmoplastic stroma with focal microcalcifications (Fig. 1D). The invasive carcinoma measured 3.5 cm and corresponded to the solid lesion identified on grossing, whereas the associated ITPN measured 6.3 cm and corresponded to the cystic lesion, with metastasis in 2 of 28 lymph nodes (pT2N1). The diagnosis was ITPN with associated carcinoma. The pancreatic resection margin was positive for ITPN with high-grade dysplasia; however, the margin was negative for invasive carcinoma. The patient received adjuvant chemotherapy with standard FOLFIRINOX including 5-FU, oxaliplatin, and irinotecan. Three months later, the dose of oxaliplatin was reduced due to neuropathy.

### Recurrence and follow-up

Seventeen months later, imaging showed a recurrent cystic lesion (10 cm) at the resection margin (Fig. 1E). Cytology confirmed recurrent carcinoma, and completion pancreatectomy with distal gastrectomy was performed. Grossly, a 13 cm cystic mass involved the pancreatic remnant with adhesions to the stomach and duodenum. Microscopically, invasive adenocarcinoma replaced nearly all residual pancreatic tissue (Fig. 1F). Following recurrence after prior adjuvant FOLFIRINOX therapy, the patient was transitioned to combined chemoradiation and a gemcitabine-based regimen consisting of gemcitabine, nab-paclitaxel, and capecitabine, followed by maintenance gemcitabine. At 2 years post–second surgery, he remains clinically stable without disease progression.

## Discussion

Here we present a case of a 30-year-old male with a pancreas carcinoma arising from ITPN. The lesion was resected with the presurgical diagnosis of chronic pancreatitis with pancreatic duct stricture and pseudocysts. Upon pathologic evaluation, ITPN with associated carcinoma was discovered. ITPN is a rare neoplasm that can occur in young adults. The age of patients with ITPN ranges from 25 to 84 years, with a mean age of 55 years [4]. Invasive carcinoma is highly associated with ITPN in up to 71% cases [4]. Thus, ITPN is a cancer precursor with a high risk of developing invasive carcinoma, and often unexpected, especially in young patients, as occurred in this reported patient. These clinical features are in contrast to the common pancreatic ductal adenocarcinoma, which has a median age of 70 years and rarely seen in patients younger than 40 years old in the United States [6].

Although carcinoma is commonly associated with ITPN, it has a significantly better prognosis with curative surgery compared to conventional pancreas ductal adenocarcinoma [7]. Five-year survival rates after surgery for ITPN-associated carcinoma have been reported up to 71% [4]. However, pre-operative diagnosis of ITPN and associated carcinoma remains challenging. Clinical presentation and imaging findings are often atypical [5], and the tumor is not recognized until pathologic examination. The presence of a solid lesion in the pancreatic duct or a cyst, larger lesion size, and main duct dilatation or stricture can suggest an intraductal neoplasm; however, these features are not always present [5]. In the present case, although direct comparison with the initial outside imaging was not possible because the studies were unavailable for review, the subsequent development of pancreatic ductal stricture and recurrent cystic lesions raised concern for an underlying neoplastic process rather than simple pseudocysts, highlighting the potential diagnostic challenge of ITPN in early presentations. Biopsy remains the diagnostic gold standard, especially when a solid lesion is visualized on imaging. In the present case, the solid component was not distinctively showed radiographically, and no biopsy was performed pre-operatively. This case suggests that in young patients with complex cystic lesions, especially when imaging findings are atypical for pseudocysts, biopsy to rule out neoplasms such as ITPN should be considered.

Although ITPN-associated invasive carcinoma generally demonstrates a more favorable prognosis compared with conventional pancreatic ductal adenocarcinoma, recurrence may still occur, particularly in cases with incomplete excision. In the present case, the presence of a positive surgical margin involved by ITPN may have contributed to tumor recurrence 17 months after the initial surgery, emphasizing the importance of complete surgical resection with negative margins whenever feasible. Nevertheless, despite recurrence, the patient remained alive after additional surgical and chemotherapeutic management, which is still consistent with the relatively improved overall survival reported for ITPN-associated carcinoma compared with conventional pancreatic ductal adenocarcinoma.

ITPN is a rare pancreatic intraductal neoplasm characterized by a predominantly tubulopapillary growth pattern, high-grade dysplasia, and minimal intracellular mucin production. Histologically, ITPNs are composed of tightly packed tubular glands and papillae lined by cuboidal to columnar cells with high-grade cytologic atypia, frequent mitotic activity, and variable intraductal necrosis. Immunohistochemically, ITPNs typically express ductal differentiation markers, including CK7, CK19, MUC1, and MUC6, while lacking or showing only minimal expression of the intestinal and gastric mucin markers MUC2 and MUC5AC. These morphologic features and immunophenotype help distinguish ITPN from IPMN, which characteristically demonstrates abundant mucin production and often expresses MUC5AC, with subtype-specific expression of MUC1 and MUC2. Morphologically, IPMNs exhibit papillary architecture with conspicuous mucin secretion and cystic dilation of pancreatic ducts, whereas ITPNs are generally more solid, exhibit a tubulopapillary configuration, and produce little grossly visible mucin. The current case has typical morphology of ITPN, showing tubularpapillary growth of glands, lack of intracellular mucin, and universal high-grade dysplasia, distinguishing it from IPMN.

The pathogenesis of ITPN and its progression to invasive adenocarcinoma are poorly understood. Molecular alterations identified to date are distinct from those in pancreatic ductal adenocarcinoma and other intraductal neoplasms, including mutations in chromatin remodeling genes (KMT2A, KMT2B, KMT2C, BAP1), PI3K pathway genes (PIK3CA, PTEN), FGFR2 fusions, and STRN-ALK fusions [8]. Unlike ductal adenocarcinoma and IPMN, ITPN lacks common mutations in KRAS, GNAS, and RNF4. Limited data suggest associated carcinomas may show TP53 mutation without KRAS, NRAS, or BRAF alterations [9].
